# Effects of shortened scanning intervals on calculated regional rates of cerebral protein synthesis determined with the L-[1-^11^C]leucine PET method

**DOI:** 10.1371/journal.pone.0195580

**Published:** 2018-04-16

**Authors:** Giampaolo Tomasi, Mattia Veronese, Alessandra Bertoldo, Carolyn Beebe Smith, Kathleen C. Schmidt

**Affiliations:** 1 Section on Neuroadaptation & Protein Metabolism, National Institute of Mental Health, Bethesda, Maryland, United States of America; 2 Department of Neuroimaging, IoPPN, King’s College London, London, United Kingdom; 3 Department of Information Engineering, University of Padova, Padova, Italy; Wayne State University, UNITED STATES

## Abstract

To examine effects of scan duration on estimates of regional rates of cerebral protein synthesis (rCPS), we reanalyzed data from thirty-nine previously reported L-[1-^11^C]leucine PET studies. Subjects consisted of 12 healthy volunteers studied twice, awake and under propofol sedation, and 15 subjects with fragile X syndrome (FXS) studied once under propofol sedation. All scans were acquired on a high resolution scanner. We used a basis function method for voxelwise estimation of parameters of the kinetic model of L-[1-^11^C]leucine and rCPS over the interval beginning at the time of tracer injection and ending 30, 45, 60, 75 or 90 min later. For each study and scan interval, regional estimates in nine regions and whole brain were obtained by averaging voxelwise estimates over all voxels in the region. In all three groups rCPS was only slightly affected by scan interval length and was very stable between 60 and 90 min. Furthermore, statistical comparisons of rCPS between awake and sedated healthy volunteers provided almost identical results when they were based on 60 min scan data as when they were based on data from the full 90 min interval. Statistical comparisons between sedated healthy volunteers and sedated subjects with FXS also yielded almost identical results when based on 60 and 90 min scan intervals. We conclude that, under the conditions of our studies, scan duration can be shortened to 60 min without loss of precision.

## Introduction

Fully quantitative measurement of regional rates of cerebral protein synthesis (rCPS) is now possible with the L-[1-^11^C]leucine positron emission tomography (PET) method. The method has been developed and validated for use in human subjects [1, 2]. The biological process of protein synthesis is important to measure because it is an essential process for growth, maintenance and function of living organisms. This is especially true in the central nervous system where *de novo* protein synthesis is involved in adaptive responses such as learning and memory and neural plasticity. Moreover, animal studies indicate that rCPS are altered in models of various clinical disorders [3–7] and in certain physiologic states such as slow-wave sleep [8]. rCPS also changes during brain development [9] and normal aging [10].

Measurements of rCPS in studies of awake age-matched healthy young men with the L-[1-^11^C]leucine PET method are reproducible and have low variability [11]. rCPS are also unaffected by propofol anesthesia in healthy young men [12]. This latter finding suggested that subjects unable to tolerate the PET scanning procedures while awake, such as those with neurodegenerative or neurodevelopmental disorders, could be studied under sedation. Such studies must be undertaken with caution, however, as the sedative agent could have an effect on rCPS in the patient group different from that seen in healthy controls [13]. An alternative strategy to increase the likelihood that patients could tolerate scanning without sedation is to reduce the total time required for scanning. Validation of the L-[1-^11^C]leucine PET method was based on a 90 min scanning interval [2]. The scanning interval of 90 min was originally chosen in order to allow sufficient time for most of the label in brain to be incorporated into [^11^C]protein; this leads to greater accuracy in estimating rCPS. However, the half-life of ^11^C is ~20 min, and the increased noise due to low count rates at the end of the study may offset the advantage of scanning for 90 min.

In the present study we examined the effects of shortened scan durations on estimates of the parameters of the leucine kinetic model and on rCPS. We found that mean regional estimates of the rate constants that act on the precursor pool of L-[1-^11^C]leucine in the tissue decreased as the length of the scan interval increased. The estimated rate constant for transport of leucine from plasma to tissue, and the estimated blood volume were only slightly altered by changes in scan interval length. Most importantly, computed values of rCPS, quantitative measurement of which is the goal of the PET scans, were very stable between 60 and 90 min.

## Materials and methods

### Data acquisition

Data from a previously-reported study of 12 healthy control subjects and 15 full mutation fragile X subjects [13] were used for reanalysis in the current study. All subjects were male and 18–24 yrs of age. Control subjects were studied once in the awake state, and on a separate day again under propofol anesthesia. Fragile X subjects were studied only under propofol sedation. The criteria for subject inclusion and the procedure for L-[1-^11^C]leucine PET studies have been described previously [13]. Studies were performed on the ECAT High Resolution Research Tomograph (HRRT) (CPS Innovations, Knoxville, TN), which has a spatial resolution of ~2.6 mm full width at half maximum (FWHM) [14]. A 90-min emission scan was initiated coincident with a 2-min intravenous infusion of 27 ± 3 mCi (mean ± SD, n = 39; range 18–31 mCi) equivalent to 1.00 ± 0.11 GBq (mean ± SD, range 0.67–1.15 GBq) of L-[1-^11^C]leucine (estimated specific activity 3 mCi/nmol (0.11 GBq/nmol)). Images were reconstructed by means of the motion-compensated 3D ordinary Poisson ordered subset expectation maximization (OSEM) algorithm [14] as 42 frames of data (16x15, 4x30, 4x60, 4x150, 14x300 sec); voxel size was 1.21x1.21x1.23 mm. Briefly, events are acquired on the HRRT in list-mode and head-motion is measured during the scan with the Polaris system [15]. Individual events are repositioned in space, and reconstruction is performed on the list-mode data by use of an OSEM-type algorithm [16]. The expected value of the number of counts is determined accounting for attenuation, normalization, randoms and scatter. Timed arterial blood samples were drawn over the course of the scan, and concentrations of total ^11^C and ^11^CO_2_ in whole blood and labeled and unlabeled leucine concentrations in arterial plasma were measured as described previously [11]. All subjects underwent a T1-weighted MRI of the brain for region of interest (ROI) placement. This study was approved by the National Institutes of Health Combined Neurosciences Institutional Review Board (06-M-0214, NCT00362843), the National Institutes of Health Radioactive Drug Research Committee, and the National Institutes of Health Radiation Safety Committee. All subjects gave written informed consent.

### Kinetic model

**Fig 1:**
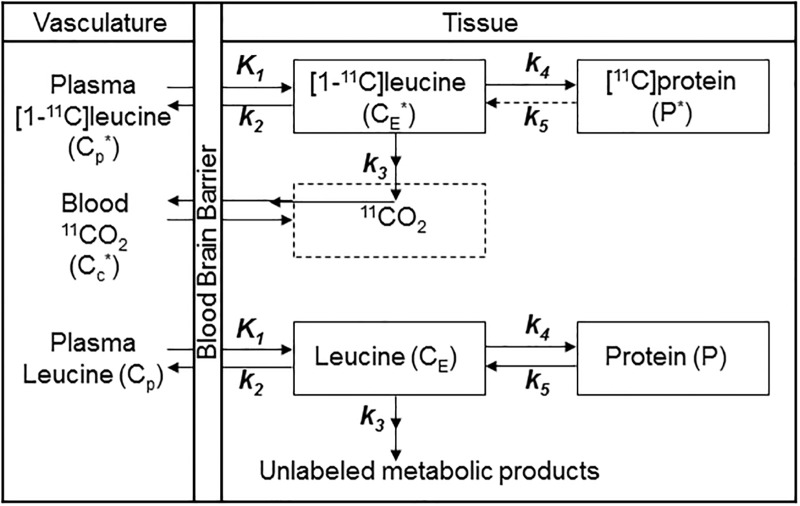
Homogeneous tissue kinetic model for L-[1-^11^C]leucine. Total concentration of ^11^C (*C*_T_*) includes free L-[1-^11^C]leucine and L-[1-^11^C]leucine incorporated into tissue protein (*C*_E_* and P*, respectively), plus activity in blood (*V*_*b*_*C*_b_*, where *V*_*b*_ is the fraction of the volume occupied by blood and *C*_b_* is the concentration of activity in whole blood). Total ^11^C also includes labeled products of L-[1-^11^C]leucine metabolism: ^11^CO_2_ and products of ^11^CO_2_ fixation. *K*_*1*_ and *k*_*2*_ are the rate constants for transport of leucine from plasma to tissue and back, respectively. *k*_*3*_ is the rate constant for the first two steps in leucine catabolism, transamination and decarboxylation, which yield ^11^CO_2_. *k*_*4*_ and *k*_*5*_ are the rate constants for leucine incorporation into protein and release of free leucine from proteolysis, respectively. It is assumed that (i) there is no significant breakdown of labeled product during the experimental interval, i.e., *k*_*5*_P*~0; (ii) fixation of ^11^CO_2_ during the experimental period is negligible [17, 18]; and (iii) equilibration of diffusible ^11^CO_2_ between brain and blood is rapid [17]. The concentration of ^11^CO_2_ in tissue is approximated by *V*_*D*_*C*_c_*, where *C*_c_* is the ^11^CO_2_ concentration in whole blood and *V*_*D*_ is the brain: blood equilibrium distribution volume of ^11^CO_2_. *V*_*D*_ was fixed at 0.41, the value measured in rhesus monkeys [2], which is in agreement with the mean whole brain: plasma equilibrium distribution volume determined from ^11^CO_2_ studies in humans [19]. The blood: plasma equilibrium distribution volume is close to unity [20]. The model assumes the tissue voxel is homogeneous with respect to concentrations of amino acids, blood flow, rates of transport and metabolism of amino acids, and rates of incorporation into protein [1]. The model used to describe labeled leucine also describes unlabeled leucine (bottom row), except that unlabeled leucine and protein are in steady state, and the steady-state breakdown of unlabeled protein (*k*_*5*_*P*) is greater than zero. Under the assumption that there is no isotope effect, the rate constants for labeled and unlabeled leucine are identical.

Fig 1 illustrates the homogeneous tissue kinetic model of the behavior of leucine in brain [1].

Based on the above kinetic model, total activity in the volume to be analyzed is given by

$$C_{T}^{*}(t)=(1-V_{b})[C_{E}^{*}(t)+P^{*}(t)+V_{D}C_{C}^{*}(t)]+V_{b}C_{b}^{*}(t).$$ (1)

Expressed in terms of rate constants we have

$$C_{T}^{*}(t;\rho )=(1-V_{b})\left\{[\frac{K_{1}(k_{2}+k_{3})}{k_{2}+k_{3}+k_{4}}]\int_{0}^{t}C_{p}^{*}(\tau )e^{-(k_{2}+k_{3}+k_{4})(t-\tau )}d\tau +[\frac{K_{1}k_{4}}{k_{2}+k_{3}+k_{4}}]\int_{0}^{t}C_{p}^{*}(\tau )d\tau +V_{D}C_{c}^{*}(t)\right\}+V_{b}C_{b}^{*}(t)$$ (2)

where *C*_p_*(*τ*) is the arterial plasma concentration of [^11^C]leucine. The dependence of total tissue activity on the parameter vector ***ρ*** = [*K*_*1*_,*k*_*2*_*+k*_*3*_,*k*_*4*_,*V*_*b*_] is now explicitly shown. Note that the parameters *k*_*2*_ and *k*_*3*_ appear only as a sum in Eq (2); they cannot, therefore, be separately identified. Eq 2 is the model equation from which the rate constants and *V*_*b*_ were estimated.

For any given tissue rCPS can be calculated from the measured plasma concentration of unlabeled leucine, *C*_*p*_, and estimated values of the rate constants as [1]:

$$rCPS=\left(\frac{K_{1}k_{4}}{k_{2}+k_{3}}\right)C_{p}$$ (3)

Eq 3 accounts for recycling of unlabeled amino acids derived from tissue protein breakdown. It may be of interest to examine separately the effects of recycling. This can be done by evaluating the factor *λ*, which represents the fraction of unlabeled leucine in the precursor pool for protein synthesis that is derived from arterial plasma; the fraction derived from tissue protein breakdown is *(1-λ)*. Lambda can be computed from the rate constants of the kinetic model as [1]:

$$\lambda =\frac{k_{2}+k_{3}}{k_{2}+k_{3}+k_{4}}$$ (4)

### Rate constant estimation

Rate constants were estimated voxelwise by use of a basis function method (BFM) that utilizes a grid search approach and linear least squares estimation [21]. In the case that any model parameter estimate is negative (or if *V*_*b*_ is >1), estimates are replaced by those from the appropriately constrained linear least squares algorithm [22]. Under the conditions of our study, i.e., high resolution data and good counting statistics, BFM analysis based on the homogeneous tissue model can be used for estimation of rCPS at the voxel level, as the impact on rCPS of kinetic heterogeneity present in some voxels in the tissue is negligible [22, 23]. The BFM grid consists of a set of values for *β* = *k*_*2*_+*k*_*3*_+*k*_*4*_ in the physiological range. For our dataset, 100 values equally spaced between *β*_min_ = 0.0001 min^-1^ and *β*_max_ = 0.5 min^-1^ were used. Weights were inversely proportional to variance in whole brain activity, which was modeled assuming Poisson statistics, as [24]

$$Var[C_{T}^{*}(t_{i})]=\alpha s_{i}^{2}=\alpha \frac{\text{exp}\left(\gamma t_{i})C_{T}^{*}(t_{i}\right)}{\Delta t_{i}}$$ (5)

where γ is the decay constant for ^11^C, *Δt*_*i*_ is the length of Frame i, *C*_T_*(*t*_*i*_*)* is the mean decay-corrected concentration of ^11^C in whole brain in Frame i, and *α* is a proportionality coefficient reflecting the noise level in the data. In PET data, *α* is not known *a priori*, but its value does not affect parameter estimates; for the parameter estimation process its value is set to one.

The difference between tracer arrival time in brain and arterial sampling site was estimated by shifting blood curves 0–30 sec, fitting the whole brain time-activity curve (from time of injection to end of scan interval under consideration), and selecting the delay that produced the smallest weighted residual sum of squares. Tracer appearance times in various parts of the brain differ from the mean of the brain as a whole by ±2 sec [25]; therefore whole brain tracer arrival delay was used for all voxels. The whole brain is very kinetically heterogeneous, not only exhibiting differences in kinetics between gray and white matter, but also within gray matter itself. Therefore, for estimating the tracer arrival delay, whole brain time-activity curves were fitted by a spectral analysis method that allows for heterogeneity within the tissue ROI [26].

Parameter estimation was performed on PET data resliced to match the subject’s MRI. Voxel size was [0.84–0.95 mm]^3^, except in one healthy volunteer whose MRI voxel size was [1 mm]^3^. Results are presented in terms of parameter values averaged over all voxels within a predefined ROI.

### Region of interest identification

ROIs were manually outlined on each subject’s MRI by visually identifying anatomic landmarks. A binary mask was constructed to identify voxels in the region. Nine ROIs and whole brain were analyzed for each subject.

To coregister MRI and PET images, a three-dimensional volume was constructed from the average of the emission data acquired between 30 and 60 min. This volume was isotropically smoothed with a Gaussian filter (FWHM 3 mm) and aligned to the MRI volume by means of the Flexible Image Registration Toolbox [27] with a 3D rigid body transformation. The resliced average 30 to 60 min PET image was visually reviewed for correct alignment with the MRI by use of Vinci software (Volume Imaging in Neurological Research, Max-Planck-Institute for Neurological Research, Cologne, Germany). The transformation parameters were then applied to each frame of the PET study (without prior smoothing) to effect their alignment with the MRI volume. After the PET data had been resliced to match the MRI, BFM was applied to estimate kinetic model parameters, *λ*, and rCPS for each voxel within the brain; estimation intervals of 0–30, 0–45, 0–60, 0–75, and 0–90 min were used. Mean parameter estimates for each ROI and each estimation interval were then computed by applying the binary ROI mask to the volumes representing voxelwise estimates of *K*_*1*_, *k*_*2*_*+k*_*3*_, *k*_*4*_, *V*_*b*_, *λ*, and rCPS. We also examined the intervoxel variability of rCPS within a region (for each region, subject, and estimation interval), by computing the coefficient of variation of the individual voxel estimates of rCPS within the ROI.

### Statistical analyses

We tested for effects of scan duration on rCPS by means of repeated measures (RM) ANOVA with RM on both regions and scan duration. Each group/condition was examined separately. In the case of a statistically significant interaction we further probed for differences by means of paired t-tests with the 90 minute value as the “control” and Dunnett’s correction for multiple comparisons.

We tested for differences in rCPS between healthy controls awake and sedated at 60 and 90 min scan durations by means of RM ANOVA (region and condition as within subjects variables). We tested for differences in rCPS between sedated healthy controls and sedated fragile subjects at 60 and 90 min scan durations by means of RM ANOVA (region as within subjects variable and diagnosis as between subjects variable). In the case of a statistically significant interaction we further probed for effects of diagnosis by means of two-tailed t-tests. Scan durations (60 and 90 min) were analyzed separately. We used SPSS, Version 21 (IBM, Armonk, NY, USA) for all statistical analyses.

## Results

### Regional estimates of kinetic model parameters and *λ*

Regional estimates of the kinetic model parameters and *λ* (means ± intersubject standard deviations (SD)) for each of the three groups are shown graphically in S1–S5 Figs. Parameters were estimated for each voxel over a 30, 45, 60, 75, or 90 min interval; for each study and interval, parameter estimates and *λ* were then averaged over all voxels in whole brain and in nine regions. Parameter estimates showed changes with increasing scan duration that were similar in all three groups, both in direction and magnitude. Estimates of *K*_*1*_ (S1 Fig) decreased slightly with increasing scan times; between the 60 and 90 min scan durations mean regional estimates decreased 3–5%. Decreases in estimates of *k*_*2*_*+k*_*3*_ (S2 Fig) and *k*_*4*_ (S3 Fig) were more substantial; between the 60 and 90 min scan durations regional estimates decreased 6–15% and 7–17% for *k*_*2*_*+k*_*3*_ and *k*_*4*_, respectively. Estimates of *V*_*b*_ (S4 Fig) increased 3–7% between 60 and 90 min. Changes in *λ* (S5 Fig) with increasing estimation interval were smaller than those in individual rate constants; the mean of the percentage decrease in *λ* was 1–3% (depending on the region) between the 60 and 90 min estimation intervals. Decreases in the estimates of *k*_*2*_*+k*_*3*_ and *k*_*4*_ with increasing estimation interval are consistent with the presence of kinetic heterogeneity in at least some voxels in the regions [22, 23, 28, 29].

### Regional estimates of rCPS

**Fig 2:**
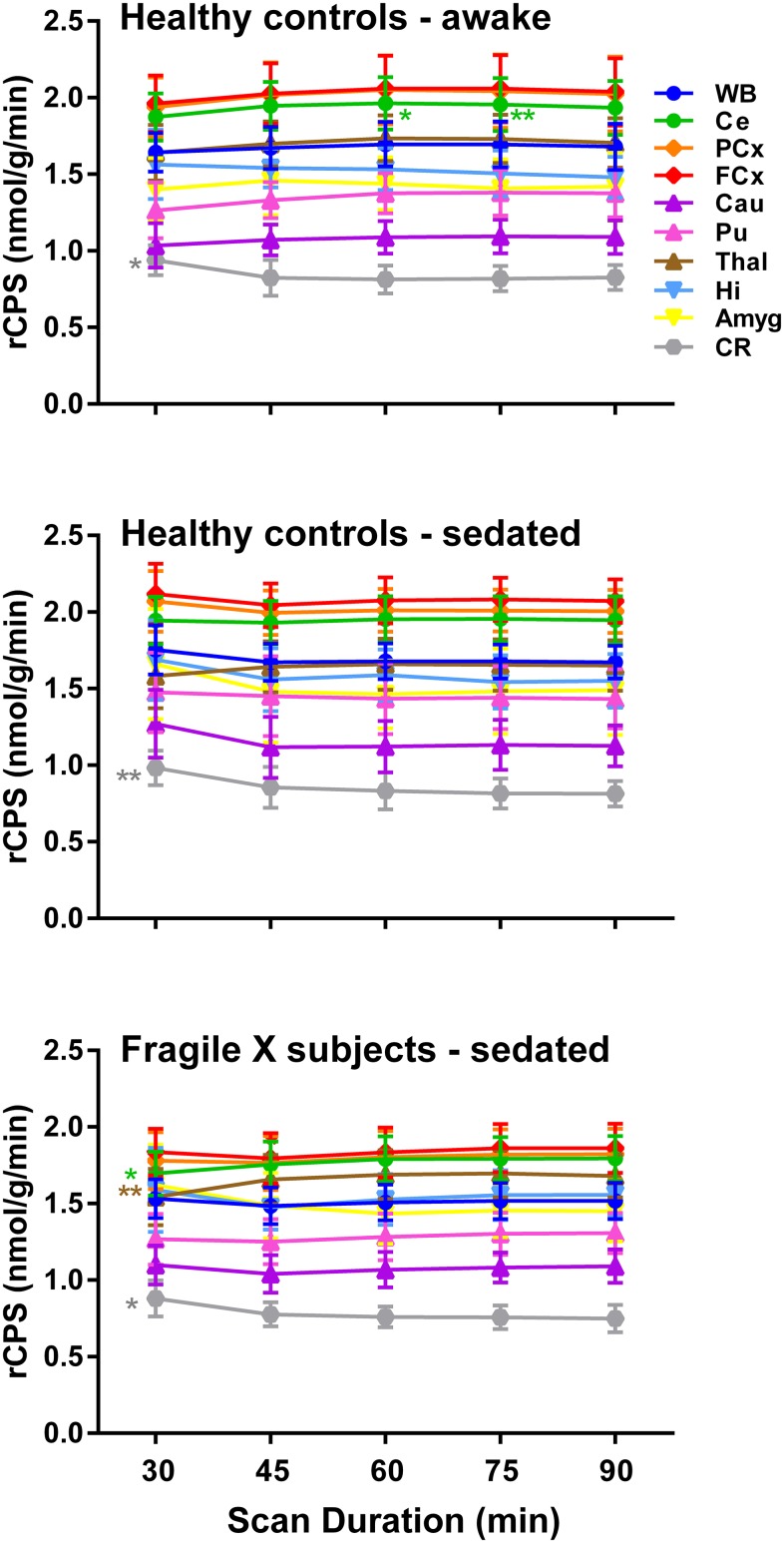
rCPS estimated over the interval beginning at the time of injection and ending 30, 45, 60, 75, or 90 min later. The Basis Function Method was used to estimate the rate constants of the homogeneous tissue kinetic model, rCPS was computed for each voxel in the brain, and ROI values were obtained by averaging values over all voxels in the ROI. The following regions were evaluated: whole brain (WB), cerebellum (Ce), frontal cortex (FCx), parietal cortex (PCx), thalamus (Thal), caudate (Cau), putamen (Pu), amygdala (Amyg), and hippocampus (Hi). Points represent means ± SD for 12 healthy control subjects studied awake, 12 healthy control subjects studied sedated, and 15 FrX subjects studied sedated. Estimates of each parameter were analyzed for statistically significant effects by means of RM ANOVA with region and scan duration as within subjects variables. **Awake healthy control group**: Interaction between region and scan duration was statistically significant (F_7.9,87.2_ = 3.82, P = 0.001). *Post-hoc* paired comparisons between the estimates at a 90 min scan duration with all other scan durations for each region are indicated on the figure. *, P ≤ 0.05; **, P ≤ 0.01. **Propofol-sedated healthy control group**: Interaction between region and scan duration was statistically significant (F_6.3,69.0_ = 2.37, P = 0.036). *Post-hoc* paired comparisons between the estimates at a 90 min scan duration with all other scan durations for each region are indicated on the figure. *, P ≤ 0.05; **, P ≤ 0.01. **Propofol-sedated subjects with FXS**: Interaction between region and scan duration was statistically significant (F_9.3,130.6_ = 4.78, P<0.001). *Post-hoc* paired comparisons between the estimates at a 90 min scan duration with all other scan durations for each region are indicated on the figure. *, P ≤ 0.05; **, P ≤ 0.01.

Regional mean estimates of rCPS for the three groups are shown in Fig 2. Differences in regional mean rCPS values over the different estimation intervals were small. The change in the mean rCPS between 60 and 90 min ranged from -3% to +2% in the awake healthy control group, from -2% to +2% in the sedated healthy control group, and from -1% to +2% in the sedated fragile X group.

**Fig 3:**
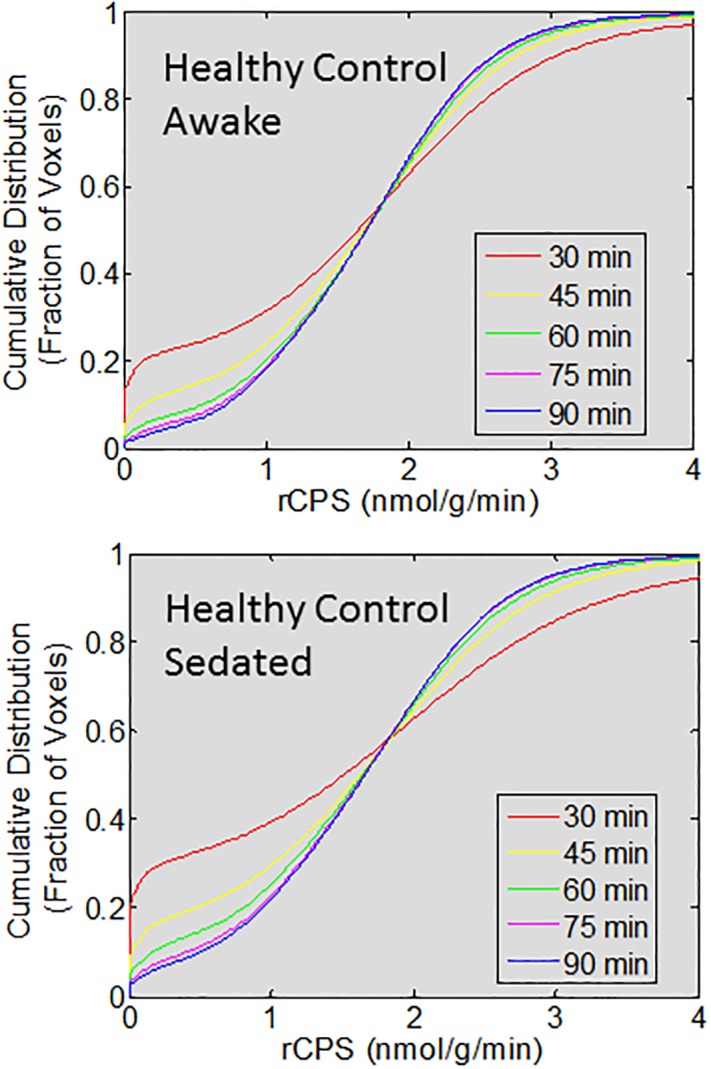
Cumulative probability distribution of rCPS. The ordinate is the fraction of the total brain voxels at each of the rCPS levels shown on the abscissa. Data are from 1.9x10^6^ voxels in the brain of a healthy 23 year old male studied awake and under propofol sedation. Values of rCPS were estimated on an interval of 30 min (red), 45 min (yellow), 60 min (green), 75 min (magenta), and 90 min (blue). rCPS was computed for each voxel in the brain from the rate constants of the homogeneous tissue kinetic model estimated with the Basis Function Method.

The empirical cumulative distributions of individual voxel values of rCPS in all brain voxels for each estimation interval are shown in Fig 3. Distributions are from studies of a single healthy control subject scanned awake and under propofol anesthesia. The data show that there are more brain voxels with rCPS not detectably different from zero when the 30 and 45 min intervals are used for estimation. There are also relatively fewer voxels with high values of rCPS with the shorter estimation intervals, most notably with the 30 min interval. The distributions of rCPS estimated on the 60, 75, and 90 min intervals were very similar except for small differences in low rCPS values. The differences in low rCPS values were slightly greater when the subject was studied under sedation.

Regional values of rCPS in whole brain and nine ROIs for all three subject groups are shown in Table 1. Values were computed from PET data with scanning durations of 60 and 90 min. Means and SDs of rCPS computed for these two scanning intervals are almost identical. The mean relative difference in rCPS did not exceed 2% in magnitude in any region or group, except in the hippocampus (Awake Control Group) and amygdala (Fragile X sedated group) where it reached 3%. In the healthy control group we compared rCPS in the awake studies with the propofol-sedated studies for each scan duration. Neither the interaction between region and condition nor the main effect of condition was statistically significant at either scan duration indicating no effect of propofol sedation on rCPS in any region.

**Table 1:** Regional rates of cerebral protein synthesis (nmol/g/min).

	60 min			
Region	Healthy volunteers conscious	Healthy volunteers sedated	Fragile X Subjects sedated	*P*-value
Whole Brain	1.70 ± 0.14	1.68 ± 0.12	1.51 ± 0.12	0.001***
Cerebellum	1.96 ± 0.17	1.95 ± 0.15	1.79 ± 0.15	0.009**
*Cortex*				
Parietal	2.05 ± 0.23	2.01 ± 0.14	1.80 ± 0.17	0.002***
Frontal	2.06 ± 0.21	2.08 ± 0.15	1.83 ± 0.16	0.001***
*Subcortical*				
Caudate	1.09 ± 0.11	1.12 ± 0.17	1.07 ± 0.12	0.340
Putamen	1.38 ± 0.13	1.43 ± 0.23	1.28 ± 0.15	0.052
Thalamus	1.73 ± 0.15	1.66 ± 0.17	1.69 ± 0.16	0.637
Hippocampus	1.53 ± 0.14	1.59 ± 0.17	1.53 ± 0.17	0.355
Amygdala	1.44 ± 0.17	1.46 ± 0.22	1.43 ± 0.20	0.741
*White Matter*				
Corona radiata	0.81 ± 0.09	0.83 ± 0.12	0.76 ± 0.07	0.065
	90 min			
Region	Healthy volunteers conscious	Healthy volunteers sedated	Fragile X Subjects sedated	*P*-value
Whole Brain	1.68 ± 0.15	1.67 ± 0.11	1.52 ± 0.12	0.002***
Cerebellum	1.93 ± 0.18	1.95 ± 0.16	1.79 ± 0.15	0.014*
*Cortex*				
Parietal	2.02 ± 0.24	2.01 ± 0.14	1.82 ± 0.17	0.005**
Frontal	2.04 ± 0.22	2.07 ± 0.14	1.86 ± 0.16	0.002***
*Subcortical*				
Caudate	1.09 ± 0.11	1.13 ± 0.13	1.09 ± 0.11	0.462
Putamen	1.38 ± 0.16	1.43 ± 0.19	1.31 ± 0.13	0.055
Thalamus	1.70 ± 0.16	1.65 ± 0.16	1.68 ± 0.14	0.621
Hippocampus	1.48 ± 0.13	1.55 ± 0.18	1.56 ± 0.14	0.923
Amygdala	1.42 ± 0.22	1.49 ± 0.29	1.45 ± 0.20	0.697
*White Matter*				
Corona radiata	0.83 ± 0.08	0.81 ± 0.08	0.75 ± 0.09	0.068

Rate constants were estimated voxelwise by use of the basis function method of Tomasi *et al* [21]. Values of regional rates of cerebral protein synthesis are the means ± SD for 12 healthy volunteers and 15 fragile X subjects, except in hippocampus and amygdala. In one healthy volunteer the quality of the MRI was poor, and we were unable to decipher the edges of these two regions; therefore, in hippocampus and amygdala data are from 11 Healthy volunteers and 15 fragile X subjects.

In the comparison of states (awake, sedated) in healthy controls at 60 min or 90 min scan durations, neither the interaction (RM ANOVA) between region and state (F_5.5,60.6_ = 1.81, P = 0.118 and F_5.0,54.8_ = 2.06, P = 0.085, respectively) nor the main effect of state (F_1,60.6_ = 0.02, P = 0.903 and F_1,54.8_ = 0.136, P = 0.720, respectively) was statistically significant, but the main effect of region was (F_5.5,60.6_ = 285, P<0.001 and F_5.0,54.8_ = 272, P<0.001, respectively). In the comparison of sedated healthy controls and fragile X subjects the interaction (RM ANOVA) between region and state was statistically significant for both the 60 min and 90 min scan durations (F_4.5,112_ = 4.70, P = 0.001 and F_4.7, 116_ = 5.89, P<0.001, respectively). Results of *post-hoc* comparisons by means of two-tailed *t*-tests comparing sedated fragile X subjects with sedated healthy volunteers are indicated in the table;

*P≤0.05;

**P≤0.01;

***P≤0.005.

We also compared rCPS in the propofol-sedated controls with the propofol-sedated fragile X subjects (Table 1). With either a 60 min or 90 min scan duration results were similar. The interaction between region and diagnosis was statistically significant at both 60 and 90 min scan durations. We further probed for statistically significant differences in specific regions by means of *post-hoc* two-tailed t-tests. Results were the same at both 60 and 90 min scan durations. In the sedated fragile X subjects rCPS was lower compared with the sedated healthy controls in the brain as a whole, cerebellum and frontal and parietal cortices (P < 0.05). Differences between healthy controls and fragile X subjects ranged from 8 to 12% regardless of the scan duration.

### Regional intervoxel variability of rCPS

**Fig 4:**
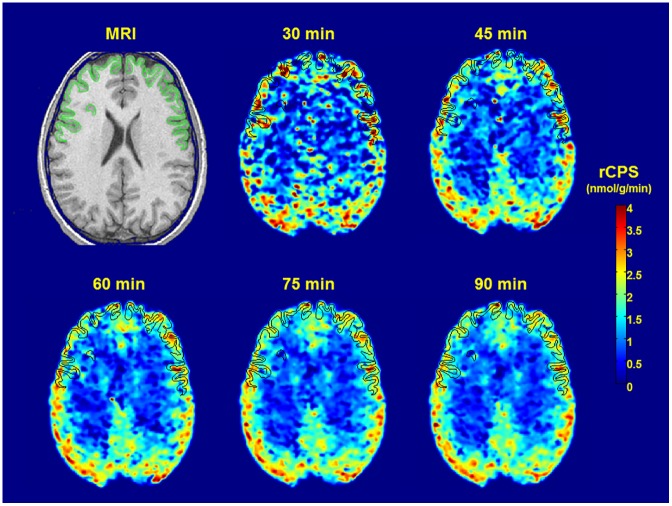
Parametric images of rates of cerebral protein synthesis (rCPS). Data from a L-[1-^11^C]leucine PET study of a healthy awake 22 year old male subject. Kinetic model parameters of the homogeneous tissue kinetic model and rCPS were estimated with the Basis Function Method over the intervals 0–30, 0–45, 0–60, 0–75, and 0–90 min. A Gaussian filter (2.0 mm FWHM kernel) was employed to smooth the parametric images in three dimensions before visualization. The MRI Image to which the PET images were coregistered is shown at the upper left. All images are in transverse plane on which the frontal cortex has been outlined.

In every subject, the intervoxel variability of rCPS within each region was observed to decrease with increasing length of the estimation interval. In whole brain the mean coefficients of variation in the awake group of control subjects were 56%, 52%, and 49% for the 60, 75, and 90 min scanning intervals, respectively. The thalamus generally had the lowest intervoxel variability (40%, 37%, and 35%) and the amygdala the highest (68%, 60%, and 54%). Interestingly, the cortical regions exhibited lower intervoxel variability than white matter (frontal cortex: 45%, 40%, and 38%; corona radiata: 64%, 54%, and 49% for the 60, 75, and 90 min scanning intervals, respectively). The other two groups of subjects showed similar results. Parametric maps of rCPS estimated on the scan intervals between 30 and 90 min in a transverse plane through the frontal cortex are shown in Fig 4.

## Discussion

Our study shows that under the conditions of high spatial resolution and voxelwise data analysis with a homogeneous tissue model, decreasing the scan duration of L-[1-^11^C]leucine PET studies from 90 to 60 min has negligible effects on estimates of rCPS.

The L-[1-^11^C]leucine PET method was developed and validated to make possible fully quantitative measurements of rCPS in human subjects [1, 2]. In order to determine rCPS, the method requires estimates of the parameters of a kinetic model that describes the time courses of [^11^C]leucine and [^11^C]protein in brain, estimates that must be made from PET measurements of total radioactivity. Immediately after tracer injection, almost all activity in brain is in the form of [^11^C]leucine. Free [^11^C]leucine in brain increases for about 5–8 min after tracer injection and then begins to clear, reaching a level of approximately 10–20% of the total activity by 90 min. The amount of [^11^C]protein, on the other hand, increases throughout the study and reaches a level of about 80–90% of the total activity by 90 min after injection. Having a scan interval that includes a period with substantial efflux of tracer from brain (early in the study) as well as a period with substantial incorporation of label into product (late in the study) enhances our ability to obtain good estimates for the kinetic model rate constants that describe these processes, and hence obtain good estimates of rCPS. While a longer tracer circulation period would, at least in theory, further improve the estimates of the kinetic model parameters and rCPS, the period cannot be extended indefinitely. Firstly, the kinetic model assumes that there is only negligible breakdown of labeled protein during the study, but eventually loss of labeled product would become significant. Secondly, ^11^C has a half-life of ~20 mins and noise due to low count rates increases significantly at later times. Finally, longer scanning times increase the discomfort for the subject who must lie as motionless as possible on the scanner bed throughout the study, and limit the number of studies that can be completed on a single scanner.

Whereas it was impractical to lengthen the scanning interval beyond 90 min, we asked whether the scan could be shortened without losing accuracy in determinations of rCPS. Shorter scanning times would be of obvious benefit to subjects, and might even make it possible to scan patients who cannot tolerate longer scanning intervals without sedation. We have now acquired a large database of L-[1-^11^C]leucine studies in which PET data were acquired over a 90 min interval. This allowed us the opportunity to investigate what would have been the results had we used a shorter scanning time.

In the present study we firstly examined the effects of shortened scan durations on estimates of the parameters of the leucine homogeneous tissue kinetic model and on rCPS by reanalyzing data from thirty-nine previously acquired PET studies. Data were analyzed over scanning intervals of 30, 45, 60, 75, and 90 min. Estimates of the kinetic model rate constants *k*_*2*_*+k*_*3*_ (for efflux from brain plus metabolism of [^11^C]leucine) and *k*_*4*_ (for incorporation of leucine into protein) were found to decrease as the length of the scanning interval increased, consistent with tissue heterogeneity in at least some voxels within each region [29]. Decreases were, however, of similar magnitude for both *k*_*2*_*+k*_*3*_ and *k*_*4*_ and their ratio, which appears in the computation of rCPS, was fairly constant. Estimates of *K*_*1*_ also decreased slightly with increasing estimation interval; this is likely due to tradeoffs made in the estimation algorithm between increases in *K*_*1*_ and decreases in *k*_*2*_*+k*_*3*_. Despite changes in estimates of individual rate constants, rCPS was substantially unchanged for estimation intervals between 60 and 90 min.

We did note a slightly smaller intervoxel variability in rCPS with increasing estimation interval. We do not know, however, if this reflects more accurate voxel estimations due to the use of the extended time course of PET data, or alternatively, if the high noise levels of the late time data actually decrease our ability to differentiate true variations in rCPS that exist at different voxel locations.

We also found that the small differences in rCPS estimated at the various scanning intervals did not alter results of the statistical comparisons between our subject condition/group. At a scanning interval of 60 min, the same regions found to be statistically significantly different between groups and even *p*-values were remarkably consistent. Thus, in all respects, we obtain the same results regardless of the analysis interval chosen.

We conclude that under the conditions of our study, i.e., high resolution voxel level data analyzed with a homogeneous tissue kinetic model, the scanning time for a L-[1-^11^C]leucine PET study can be reduced from 90 to 60 min without loss of precision.

## Supporting information

S1 Dataset Individual values for the estimated parameters and rCPS. (XLSX)

S1 Fig *K*_*1*_, the rate constant for transport of leucine from plasma to tissue, estimated over the interval beginning at the time of injection and ending 30, 45, 60, 75, or 90 min later. The Basis Function Method, based on the homogeneous tissue kinetic model for L-[1-^11^C]leucine, was used to estimate *K*_*1*_ for each voxel in the brain, and ROI values were obtained by averaging estimates over all voxels in the ROI. The following regions were evaluated: whole brain (WB), cerebellum (Ce), frontal cortex (FCx), parietal cortex (PCx), thalamus (Thal), caudate (Cau), putamen (Pu), amygdala (Amyg), and hippocampus (Hi). Points represent means ± SD for 12 healthy control subjects studied awake, 12 healthy control subjects studied sedated, and 15 FrX subjects studied sedated.
(TIF)

S2 Fig *k*_*2*_*+k*_*3*_, the rate constant for transport of leucine from tissue to plasma plus the rate constant for the first two steps in leucine catabolism, estimated over the interval beginning at the time of injection and ending 30, 45, 60, 75, or 90 min later. The Basis Function Method, based on the homogeneous tissue kinetic model for L-[1-^11^C]leucine, was used to estimate *k*_*2*_*+k*_*3*_ for each voxel in the brain, and ROI values were obtained by averaging estimates over all voxels in the ROI. The following regions were evaluated: whole brain (WB), cerebellum (Ce), frontal cortex (FCx), parietal cortex (PCx), thalamus (Thal), caudate (Cau), putamen (Pu), amygdala (Amyg), and hippocampus (Hi). Points represent means ± SD for 12 healthy control subjects studied awake, 12 healthy control subjects studied sedated, and 15 FrX subjects studied sedated.
(TIF)

S3 Fig *k*_*4*_, the rate constant for leucine incorporation into protein, estimated over the interval beginning at the time of injection and ending 30, 45, 60, 75, or 90 min later. The Basis Function Method, based on the homogeneous tissue kinetic model for L-[1-^11^C]leucine, was used to estimate *k*_*4*_ for each voxel in the brain, and ROI values were obtained by averaging estimates over all voxels in the ROI. The following regions were evaluated: whole brain (WB), cerebellum (Ce), frontal cortex (FCx), parietal cortex (PCx), thalamus (Thal), caudate (Cau), putamen (Pu), amygdala (Amyg), and hippocampus (Hi). Points represent means ± SD for 12 healthy control subjects studied awake, 12 healthy control subjects studied sedated, and 15 FrX subjects studied sedated.
(TIF)

S4 Fig *V*_*b*_, the fraction of blood in the tissue, estimated over the interval beginning at the time of injection and ending 30, 45, 60, 75, or 90 min later. The Basis Function Method, based on the homogeneous tissue kinetic model for L-[1-^11^C]leucine, was used to estimate *V*_*b*_ for each voxel in the brain, and ROI values were obtained by averaging estimates over all voxels in the ROI. The following regions were evaluated: whole brain (WB), cerebellum (Ce), frontal cortex (FCx), parietal cortex (PCx), thalamus (Thal), caudate (Cau), putamen (Pu), amygdala (Amyg), and hippocampus (Hi). Points represent means ± SD for 12 healthy control subjects studied awake, 12 healthy control subjects studied sedated, and 15 FrX subjects studied sedated.
(TIF)

S5 Fig *λ*, the fraction of unlabeled leucine in the precursor pool for protein synthesis that is derived from arterial plasma, estimated over the interval beginning at the time of injection and ending 30, 45, 60, 75, or 90 min later. Based on parameter estimates of *k*_*2*_*+k*_*3*_ and *k*_*4*_, *λ* was computed for each voxel in the brain, and ROI values were obtained by averaging values over all voxels in the ROI. The following regions were evaluated: whole brain (WB), cerebellum (Ce), frontal cortex (FCx), parietal cortex (PCx), thalamus (Thal), caudate (Cau), putamen (Pu), amygdala (Amyg), and hippocampus (Hi). Points represent means ± SD for 12 healthy control subjects studied awake, 12 healthy control subjects studied sedated, and 15 FrX subjects studied sedated.
(TIF)
